# Supplementary material for: Circumsporozoite Protein of Plasmodium berghei- and George Baker Virus A-Derived Peptides Trigger Efficient Cell Internalization of Bioconjugates and Functionalized Poly(ethylene glycol)-b-poly(benzyl malate)-Based Nanoparticles in Human Hepatoma Cells
Source: Pharmaceutics. 2022 Apr 6;14(4):804. doi: 10.3390/pharmaceutics14040804 (PMC9028075; doi:10.3390/pharmaceutics14040804)
Supplement: Supplementary file 1 [file pharmaceutics-14-00804-s001.zip › pharmaceutics-1653291-supplementary.pdf]

pharmaceutics

MDPI

Supplementary Materials: Circumsporozoite Protein of *Plasmodium berghei*- and George Baker Virus A-Derived Peptides Trigger Efficient Cell Internalization of Bioconjugates and Functionalized Poly(ethylene glycol)-*b*-poly(benzyl malate)-Based Nanoparticles in Human Hepatoma Cells

Elise Vène, Kathleen Jarnouen, Catherine Ribault, Manuel Vlach, Yann Verres, Mickaël Bourgeois, Nicolas Lepareur, Sandrine Cammas-Marion and Pascal Loyer

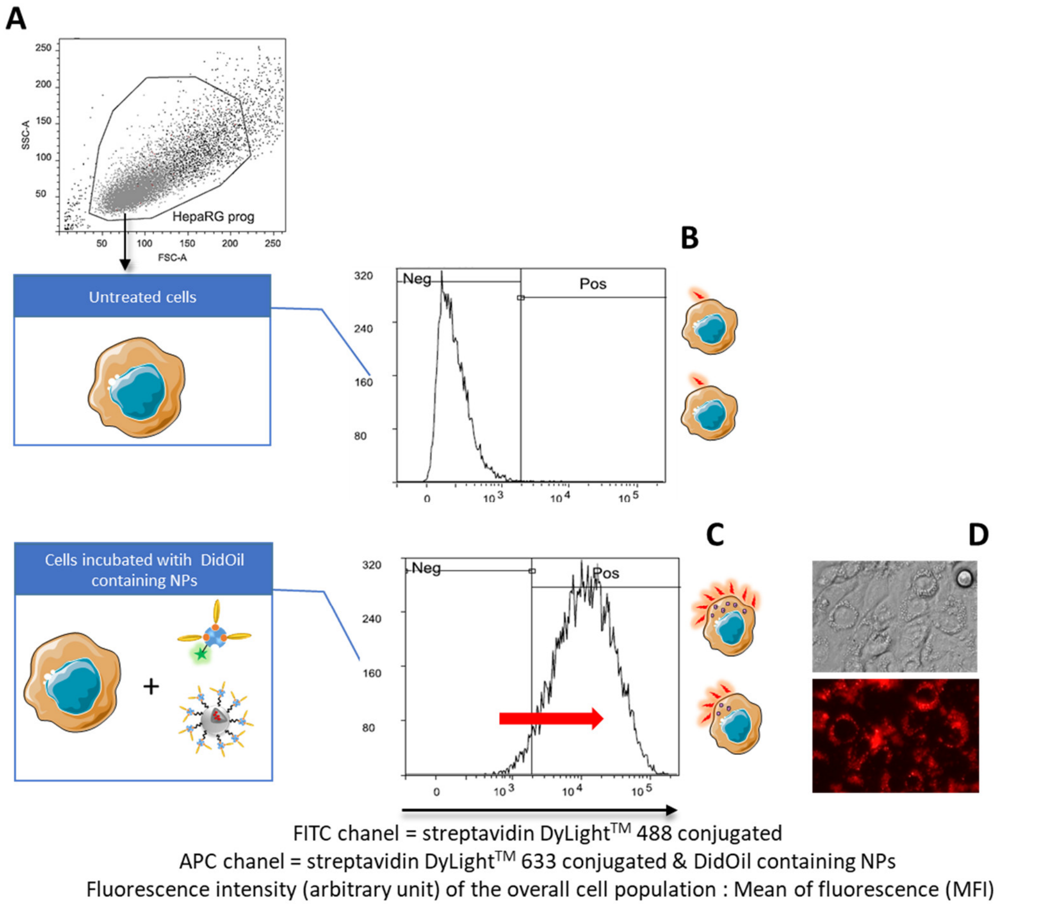

Figure S1. The cells analyzed by FACS were gated using a dot plot with the SSC (granularity: y) and FSC (size: x) parameters (**A**). Then, cells from this first population were further gated using the SSC-Height versus SSC-Area to isolate the single cells (not shown). From the single cell population, fluorescence was analyzed ((**B**)-upper histogram : fluorescence on x axis versus cell counts on y axis) in cells that were not incubated with fluorescent NPs or streptavidin to define the "background" fluorescence also called auto-fluorescence of negative cells (Neg). Then, the cells incubated with the different fluorescent NPs or bioconjugates were analyzed ((**C**)-lower histogram), which defined the positive cells (Pos) that internalized the fluorescent probes. The values of MFI presented in this work represent the overall fluorescence of all the single cells (Neg + Pos). Prior detachment of cells for flow cytometry analysis, fluorescent NPs can be visualized by confocal microscopy in cells ((**D**)-HepaRG cells containing fluorescent NPs).

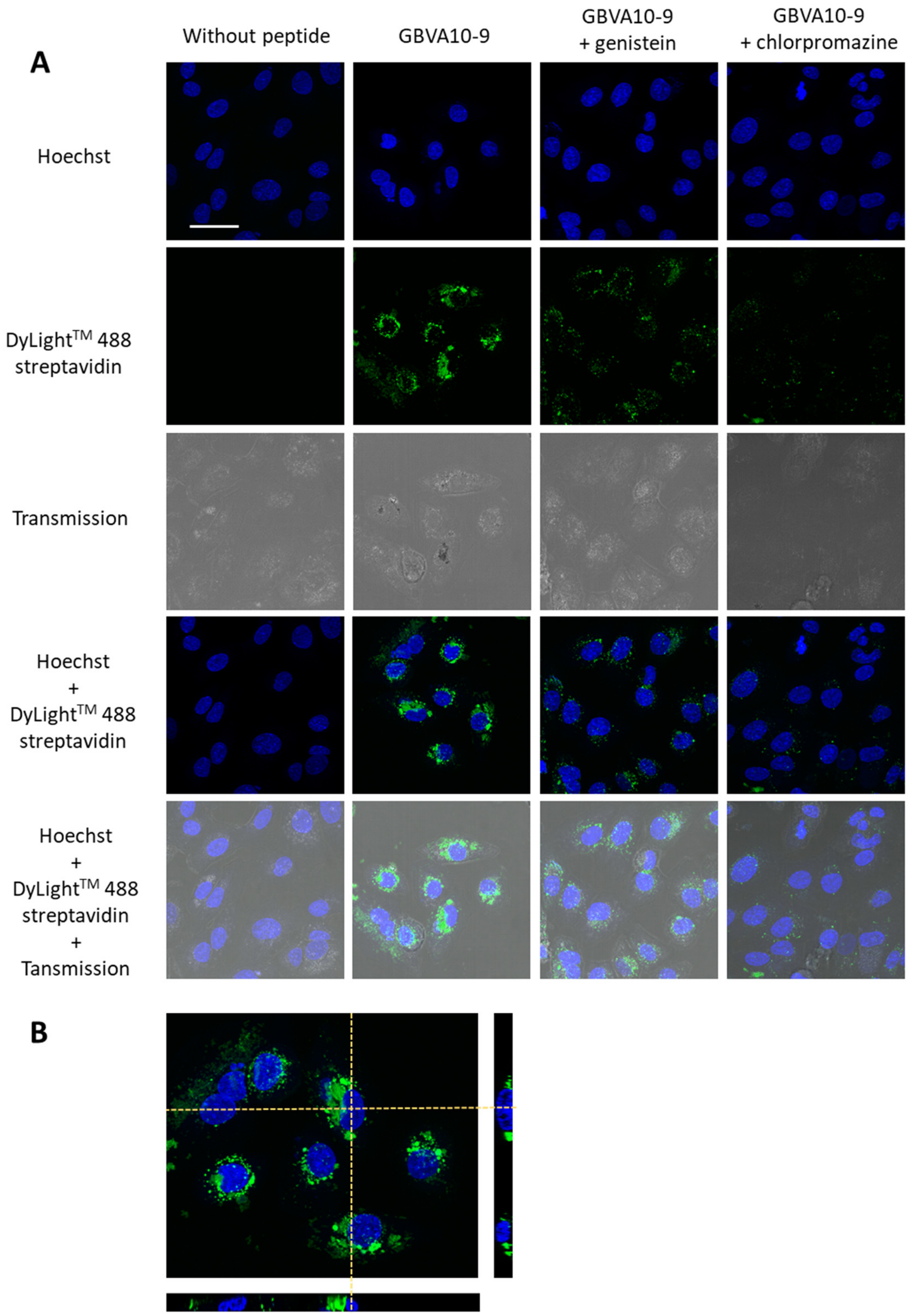

Figure S2. Intracellular detection of peptide- streptavidin DyLight 488® conjugates. (**A**) Confocal microscopy images of progenitor HepaRG cells after 24 hours incubation with streptavidin DyLight 488® conjugated to biotin (without peptide) and biotinylated-GBVA10-9 peptide in absence or presence of endocytosis inhibitors genistein and chlorpromazine. Fluorescent streptavidin DyLight 488® (green) and the nuclear DNA stained with Hoechst (blue) detected separately and presented as the merge of the double staining and the transmission light. (**B**) High magnification of HepaRG cells incubated with biotinylated-GBVA10-9 peptide streptavidin DyLight 488® conjugate with orthogonal plan. Bar corresponds to 50 µm.

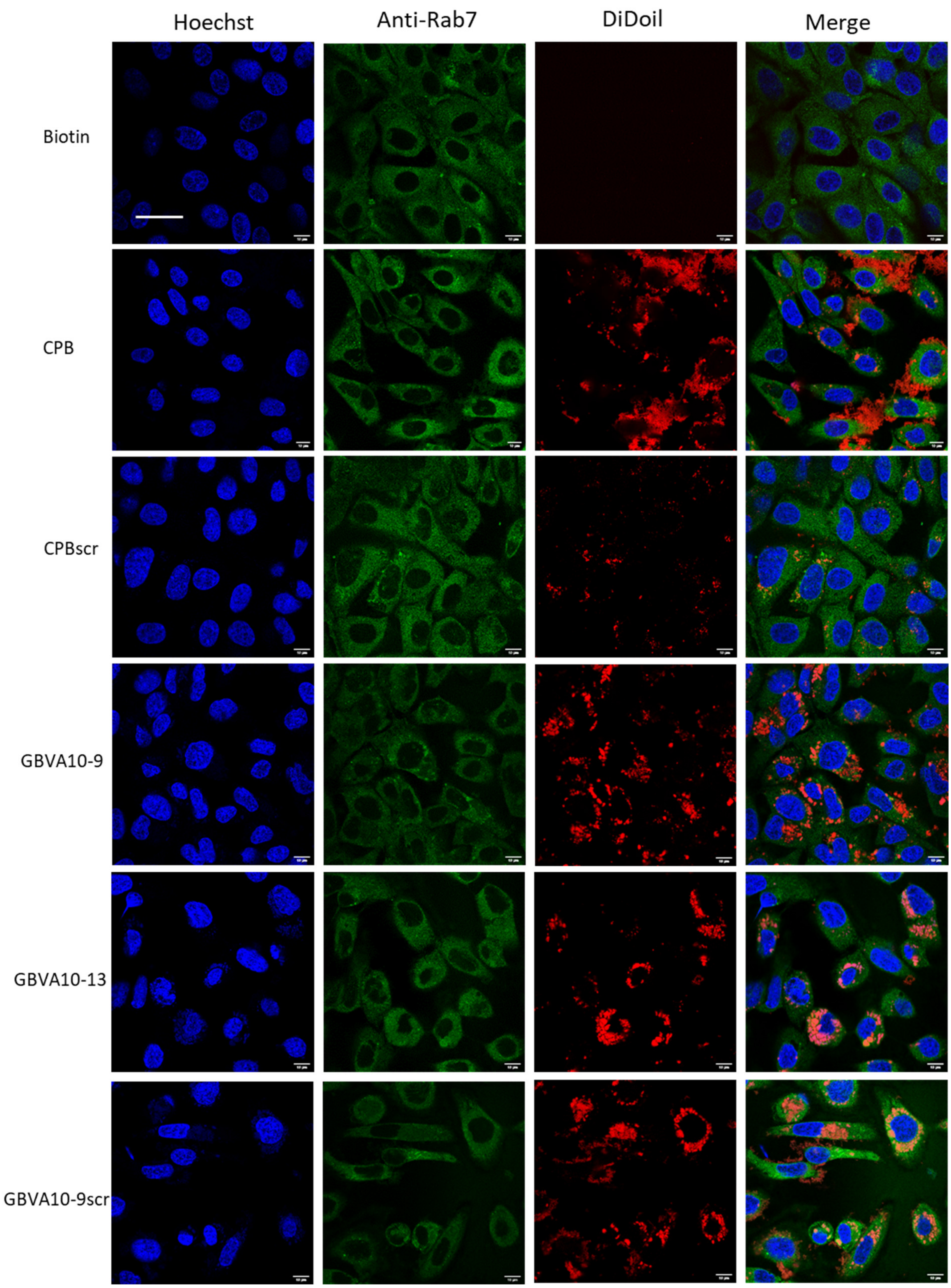

Figure S3. Colocalization of Rab7 GTPase and fluorescent NPs. Confocal microscopy of progenitor HepaRG cells incubated with CPB, GBVA10-9 and their modified (mutated/scrambled) peptides for the detection of the endosomal Rab7 GTPase (green), fluorescent nanocarriers containing DiD Oil (red) and the nuclear DNA stained with Hoechst (blue) detected separately and presented as the merge of the triple staining. Bar corresponds to 50 μm.

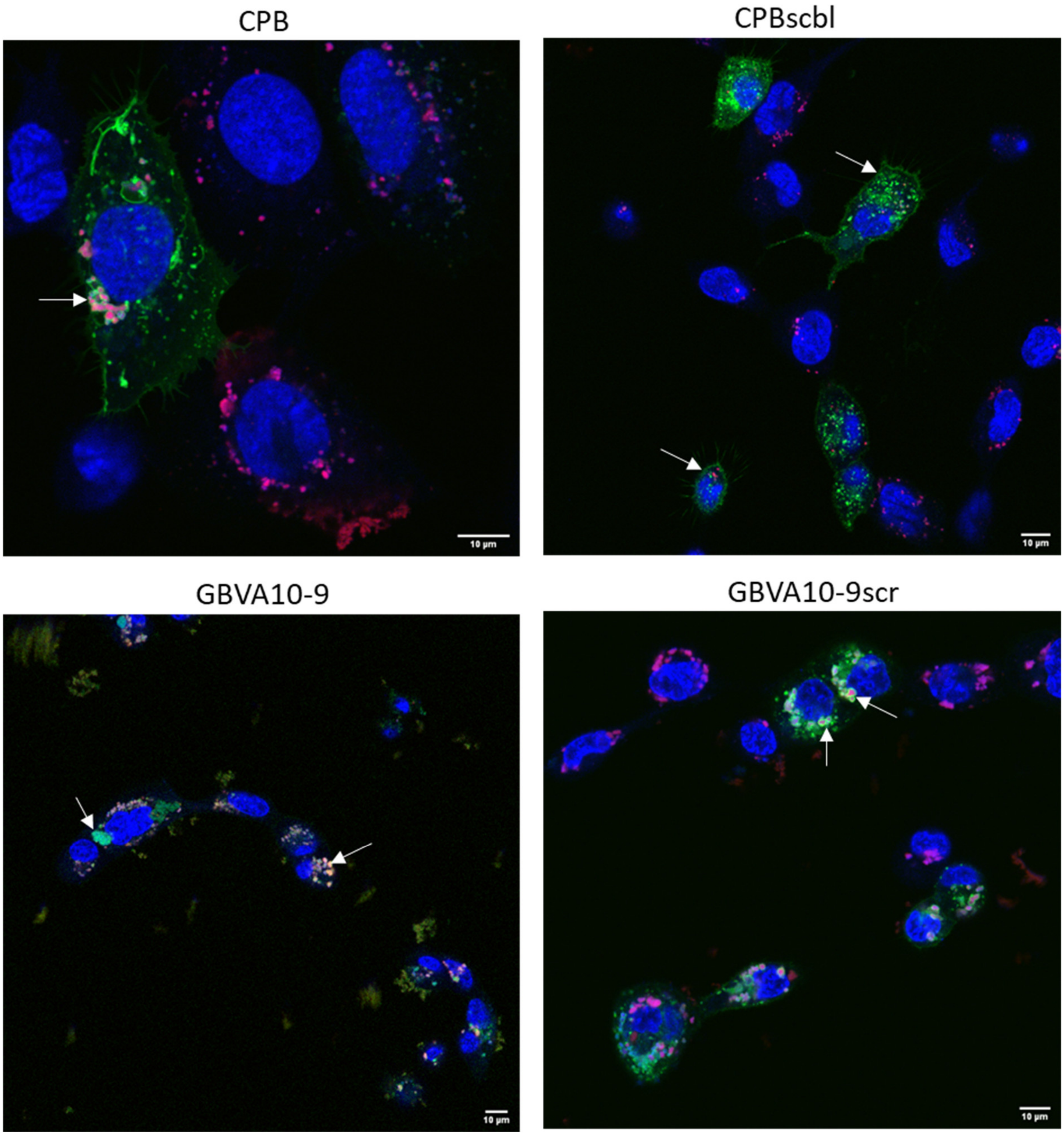

Figure S4. Colocalization of Lysosome-associated membrane protein 1 (LAMP1) protein and GBVA10-9-decorated fluorescent NPs. Confocal microscopy allowing the visualization of the strong cytoplasmic accumulation of DiD Oil-containing NPs around cell nuclei, particularly for GBVA10-9- and GBVA10-9scr-decorated NPs. The DiD Oil staining partially co-localized with the lysosomes expressing the Lysosome-associated membrane protein 1 (LAMP1)-Green fluorescent fusion protein.

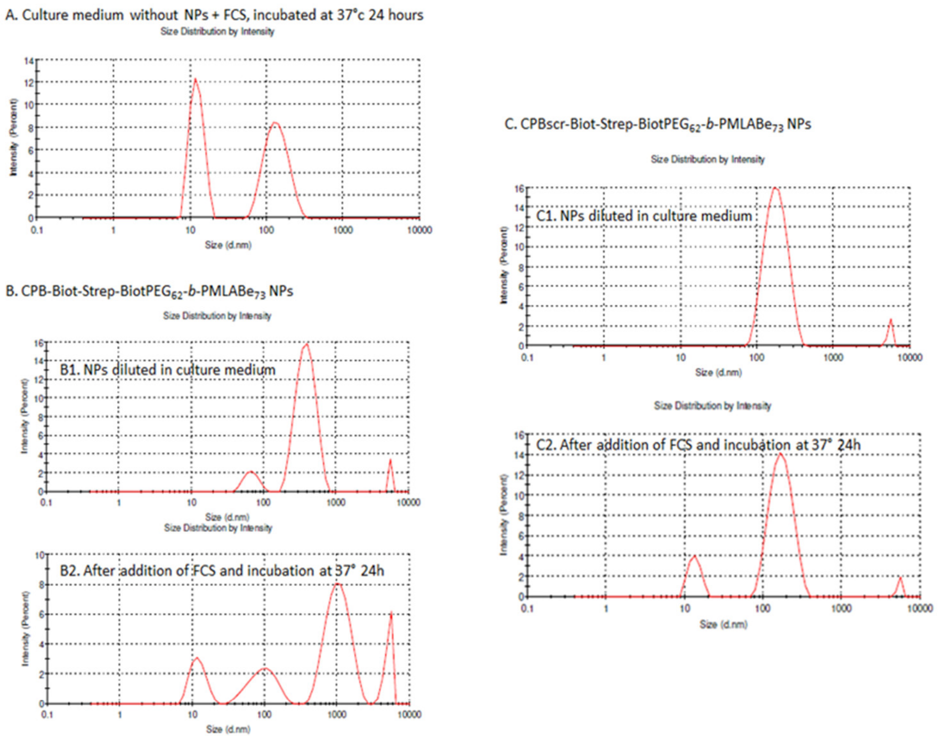

D

	NPs Suspension diluted in culture medium			NPs suspension + FCS + Incubation 37°C/24h		
Culture medium and NPs	Dh Major peak (nm)	Dh Minor peaks (nm)	Zeta potential (mV)	Dh Major peak (nm)	Dh Minor peaks (nm)	Zeta potential (mV)
Medium without NPs	/	/	/	10 (47%)<br>140 (53%)	/	- 9
$BiotPEG_{62}$-b-$PMLABe_{73}$	70 (63%)	1 (22%)<br>3,300 (15%)	- 3	100 (36%)	10 (23%)<br>540 (28%)<br>5,000 (13%)	- 8
Biot-Strept-$BiotPEG_{62}$-b-$PMLABe_{73}$	120 (100%)	/	- 6	110 (77%)	10 (18%)<br>4,300 (5%)	- 9
CPB-Biot-Strept-$BiotPEG_{62}$-b-$PMLABe_{73}$	390 (87%)	70 (9%)<br>5,600 (4%)	- 3	1,100 (57%)	10 (14%)<br>100 (19%)<br>5,000 (10%)	- 5
CPBscr-Biot-Strept-$BiotPEG_{62}$-b-$PMLABe_{73}$	180 (97%)	5,400 (3%)	- 3	180 (84%)	10 (14%)<br>5,400 (2%)	- 6
GBVA10-9-Biot-Strept-$BiotPEG_{62}$-b-$PMLABe_{73}$	1,380 (100%)	/	- 5	2,800 (89%)	360 (10%)<br>5,500 (1%)	- 7
GBVA10-13-Biot-Strept-$BiotPEG_{62}$-b-$PMLABe_{73}$	390 (92%)	40 (2%)<br>4,800 (6%)	- 4	980 (78%)	10 (7%)<br>70 (8%)<br>5,000 (7%)	- 6
GBVAscr-Biot-Strept-$BiotPEG_{62}$-b-$PMLABe_{73}$	1,170 (100%)	/	- 4	1,300 (78%)	10 (7%)<br>40 (6%)<br>5,000 (9%)	- 7
CTRLneg19-Biot-Strept-$BiotPEG_{62}$-b-$PMLABe_{73}$	1,000 (100%)	/	- 4	1,700 (70%)	10 (5%)<br>40 (5%)<br>4,700 (20%)	- 6

Figure S5. Characterization of the size distribution of NPs functionalized with peptides in culture media. Size distribution by intensity graphs obtained by DLS measurement in (**A**) culture medium without NPs containing fetal calf serum (FCS) incubated at 37 °C during 24 h, (**B**) culture medium containing CPB-Biot-Strept-BiotPEG62-b-PMLABe73 NPs before (**B1**) and after (**B2**) incubation at 37 °C for 24 h in presence of FCS, (**C**) culture medium containing CPBscr-Biot-Strept-BiotPEG62-b-PMLABe73 NPs before (**C1**) and after (**C2**) incubation at 37 °C for 24 h in presence of FCS. These incubations of NPs in culture media were performed in absence of cells. (**D**) DLS and ELS characteristics of culture media without NPs and with NPs (control and peptide functionalized NPs) before (left column) and after (right column) incubation at 37 °C in presence of FCS: values of the hydrodynamic diameter (Dh in nm) and zeta potential (in mV) of the control and peptide functionalized NPs. While zeta potential is not modified during the incubation at 37 °C, Dh of CPB- and GBVA10-9 functionalized NPs are much higher than that of NPs without peptides and these Dh increased during incubation at 37 °C in presence of FCS.

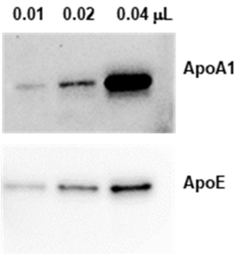

Figure S6. Characteristics of HDL purified from human serum. Immunoblotting of ApoA1 and ApoE in purified human HDL.

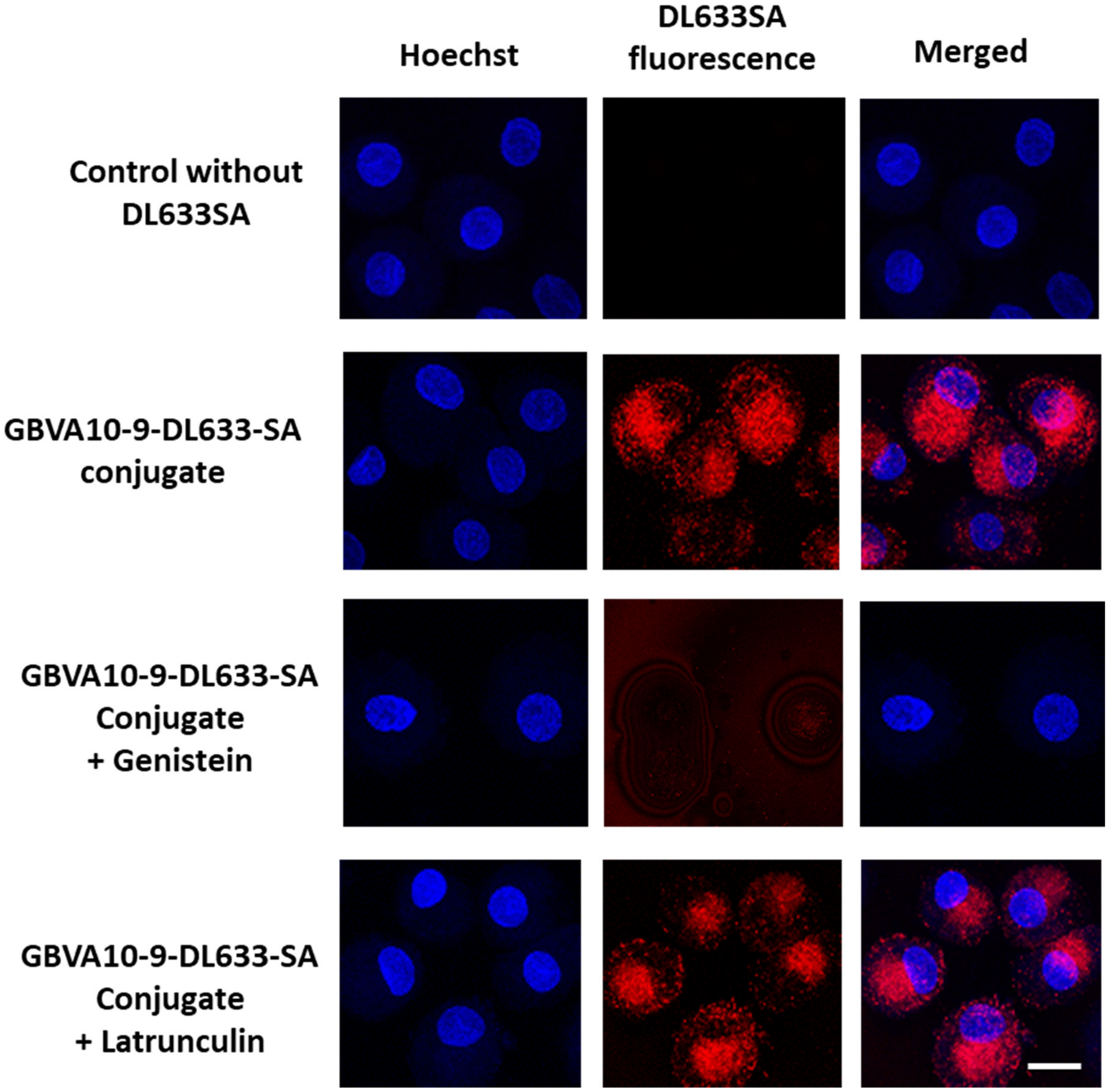

Figure S7. Confocal microscopy allowing the visualization of GBVA10-9-DL633-SA conjugates in human macrophages (HPM) in pure cultures (without Huh7 hepatoma cells) in absence or presence of phagocytosis inhibitor (latrunculin 500 nM) or endocytosis inhibitor (genistein 100 µM). Nuclear DNA was stained with Hoechst (blue) detected separately and presented as the merge of the red fluorescence of the DL633-SA. Bar corresponds to ~10 µm.
